# Diseases of the Eye

**Published:** 1898-09

**Authors:** 


Diseases of the Eye. By Edward Nettleship, F. R. C. S., Ophthalmic Surgeon at St. Thomas' Hospital, London; Surgeon to the Royal London (Moorfields) Ophthalmic Hospital. Revised and edited by W. T. Holmes Spicer, M. A., M. B., F. R. C. S., Ophthalmic Surgeon to the Metropolitan Hospital and to the Victoria Hospital for Children. With a supplement on Color Blindness by William Thompson, M. D., Emeritus Professor of Ophthalmology in the Jefferson Medical College of Philadelphia. Handsome 12mo of 521 pages, with 2 colored plates and 161 engravings. Cloth, $2.25. Lea Brothers & Co., Publishers, Philadelphia and New York.

As a text-book for students and for the purpose of ready reference, this book has long been a favorite. It is a useful book for students, general practitioners, and those making a specialty of eye work. As an elementary treatise, it is probably the best work on the subject of diseases of the eye. The present edition, the sixth, was edited by W. T. Holmes Spicer, M. A., M. B., and in this revision the book has been brought down to the present time, giving the latest and most important knowledge on this branch. A supplement has been added on "The practical examination of railway employes as to color blindness, acuteness of vision and hearing," by Prof. William Thompson, of Philadelphia. This supplement adds considerably to the value of the book. S. E. H.

